# Investigation of the Effects of Non-Conjugated Co-Grafts on the Spectroelectrochemical and Photovoltaic Properties of Novel Conjugated Graft Copolymers Based on Poly(3-hexylthiophene)

**DOI:** 10.3390/polym10101064

**Published:** 2018-09-25

**Authors:** Tomasz Jarosz, Karolina Gebka, Kinga Kepska, Mieczyslaw Lapkowski, Przemyslaw Ledwon, Pawel Nitschke, Agnieszka Stolarczyk

**Affiliations:** 1 Department of Physical Chemistry and Technology of Polymers, Silesian University of Technology, 9 Strzody Street, 44-100 Gliwice, Poland; tomasz.jarosz@polsl.pl (T.J.); karolina.gebka@polsl.pl (K.G.); kinga.kepska@polsl.pl (K.K.); mieczyslaw.lapkowski@polsl.pl (M.L.); przemyslaw.ledwon@polsl.pl (P.L.); 2 Department of Inorganic Chemistry, Analytical Chemistry and Electrochemistry, Silesian University of Technology, 6 Krzywoustego Street, 44-100 Gliwice, Poland; 3 Centre of Polymer and Carbon Materials Polish Academy of Sciences, 34 Curie-Sklodowskiej Street, 41-819 Zabrze, Poland; pnitschke@cmpw-pan.edu.pl

**Keywords:** poly(3-hexylthiophene), graft copolymers, poly(ethylene glycol), functionalized conducting polymers, solar cells

## Abstract

A new type of polysiloxane copolymers, with conjugated–regioregular poly(3-hexylthiophene) (P3HT) and non-conjugated-poly(ethylene glycol) (PEG)-grafts have been synthesised, and their properties have been studied alongside those of the parent conjugated polymer (P3HT). Spectroelectrochemical and conductometric analyses revealed an early rise of the conductance of the polymers. Once spectral changes begin taking place, the conductance is stable, implying a loss of mobility of charge carriers, even though standard doping/dedoping patterns are observed. Prototype bulk heterojunction solar cells have been fabricated, based on P3HT/[6,6]-Phenyl-C61-butyric acid methyl ester (PCBM), as well as by substituting P3HT for each of the copolymers. The prototype solar cells achieved PCEs of up to 2.11%. This is one of the highest reported power conversion efficiency (PCE) for devices based on P3HT with low average molecular weight *M*_n_ = 12 kDa. Strong correlation between the structure of the copolymer and its photovoltaic performance was found. Elongation of PEG copolymer chain and the use of methyl group instead of terminal hydroxyl groups significantly improved photovoltaic performance.

## 1. Introduction

Organic semiconductors, due to their processability, corrosion resistance and cost-efficiency being more favourable than those of inorganic materials, find applications in many optoelectronic devices, such as solar cells, light emitting diodes and field-effect transistors [1]. Solar cells are a particularly important type of devices, as they constitute a renewable source of energy. Currently, a composite electron donor/electron acceptor system of poly(3-hexylthiophene) (P3HT)/phenyl-C_61_-butyric acid methyl ester (PCBM) is the benchmark of organic solar cell materials [2], offering good device efficiency and relatively long device lifetimes. Although more sophisticated organic/polymeric systems have been developed, rivalling or even exceeding P3HT/PCBM-based solar cells in terms of efficiency [3], their drawbacks are often complex synthetic procedures and the requirement of exceedingly expensive reagents. The search for new organic solar cell materials, as evidenced by the multitude of reports [4] being published each year. That said, what if it were possible to further improve the performance of P3HT?

P3HT/PCBM-based solar cells often suffer decreased efficiency due to common issues related to organic photovoltaics [5]. Different issues were identified [6]. The main reason is possibly photo-oxidation of P3HT in the presence of oxygen. Photodegradation under ultraviolet (UV) exposure is another important process leading to degradation. It is caused by light-induced breaking of C–H bonds [7,8] However, if devices are well protected from oxygen and UV exposure, P3HT is chemically stable [9,10]. Another critical factor is morphology of the BHJ, affected adversely by, for example phase separation [11]. One of the strategies to improve morphology is to increase the ordering of the material, so as to improve the photovoltaic performance [12].

Consequently, a modification of the structure of P3HT—a dilution of the polymer on a molecular scale, can serve to improve the performance of such solar cells. One solution for achieving such molecular dilution is to graft P3HT chains on a molecular scaffold, in order to increase the inter-chain distances beyond what is typically found for P3HT. Polysiloxanes are a favourable candidate for this role, due to their good adhesion to glass and indium–tin oxide (ITO) [13,14], their relative cost-efficiency, and the ease of their modification [15].

Among various polysiloxanes, poly(methylhydrosiloxane) (PMHS) is an interesting choice, as it can be readily modified with P3HT and other grafts, due to the reactivity of the silicon–hydrogen bond [16] via the hydrosilylation method [17]. Therefore, only a slight modification (as readily introduced in the synthesis of P3HT by the McCullough method [18,19,20]) is needed to produce a polysiloxane grafted with P3HT chains. The advantages of this method, apart from its one pot feasibility, are that we can readily control the grafting density of P3HT and introduce both inert and active *co*-grafts, as desired [21].

In this manuscript, we explore the possibility of improving the parameters of solar cells based on the P3HT/PCBM composite by substituting P3HT with a series of graft copolymers. As a proof of concept, apart from grafting P3HT, we have also *co*-grafted poly(ethylene glycol) (PEG) chains onto the polysiloxane scaffold. Several types of PEG, varying in terms of their molecular weight and terminal groups have been used for this purpose, allowing us to closely examine the effects of the non-conjugated *co*-grafts on the parameters of solar cells based on the composite of our copolymers and PCBM.

## 2. Materials and Methods

### 2.1. Materials

2,5-dibromo-3-hexylthiophene ≥97%, TCI (Tokyo, Japan), [1,3-bis(diphenylphosphino)-propane]dichloronickel(II) (Ni(dppp)Cl_2_, ≥97% Sigma Aldrich, St. Louis, MO, USA), vinylmagnesium bromide (0.7 M solution in tetrahydrofuran, Acros Organics (Geel, Belgium)), *tert*-butylmagnesium chloride (2 M solution in diethyl ether, Sigma Aldrich), anhydrous tetrahydrofuran (99.9%, Acros organics) distilled before use, poly(methylhydrosiloxane) trimethylsilyl terminated (average *M*_n_ = 390, Sigma Aldrich (Saint Louis, MO, USA)), poly(ethylene glycol) methyl ether methacrylate (average *M*_n_ = 500, *M*_n_ = 950 and *M*_n_ = 300, Sigma Aldrich), poly(ethylene glycol) methacrylate (average *M*_n_ = 500, Sigma Aldrich), platinum(0)-1,3-divinyl-1,1,3,3-tetramethyldisiloxane (PTDD, Karstedt’s catalyst) (complex solution in xylene, Pt ~ 2%, Sigma Aldrich), anhydrous toluene (99.85%, Acros Organics).

### 2.2. Syntheses

**Scheme 1:**
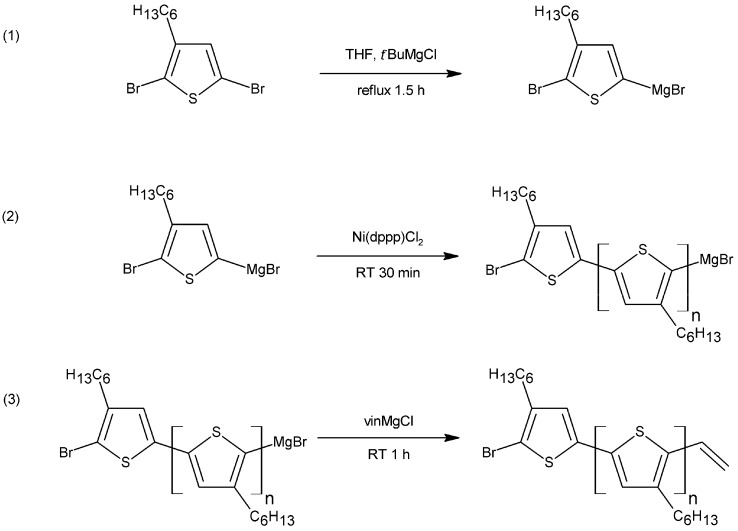
Synthesis scheme of vinyl terminated regioregular poly(3-hexylthiophene) (P3HTvin) via the Grignard metathesis method (GRIM) procedure.

Synthesis of rrP3HT vinyl terminated (P3HTvin) was carried out via the Grignard metathesis method (GRIM) method [19], as presented in Scheme 1.

To the three-necked flask anhydrous tetrahydrofuran (THF), monomer (2,5-dibromo-3-hexylthiophene) and *t*-butylmagnesium chloride were added, using a syringe through the septum, and the metathesis reaction was carried out for 1.5 h at reflux. After that the flask has been cooled down to room temperature and Ni(dppp)Cl_2_ was added. Polymerization was carried out for 30 min in room temp. Next, vinylmagnesium bromide was added and reaction was continued for 1 h. The crude product was precipitated in methanol and purified by continuous extraction using: methanol, hexane and chloroform. The obtained compound had a molecular weight of approx. 12,000 g/mol and a dispersity of 1.4 (determined by size-exclusion chromatography).

**Scheme 2:**
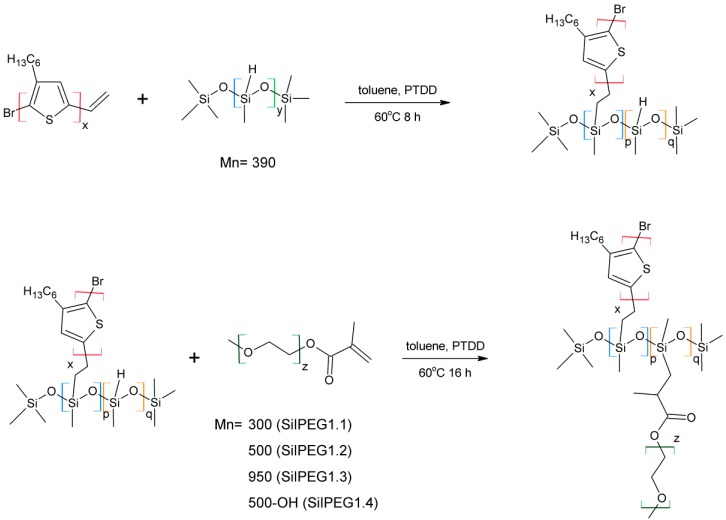
The synthetic route to poly(methylhydrosiloxane)-graft-[poly(3-hexylthiophene); poly(ethylene glycol)]s (SilPEG).

Graft copolymers we obtained using hydrosilylation reaction based on the Ganicz research group method [22], as depicted in Scheme 2.

To the three-necked flask, P3HTvin and toluene were added, and the flask was heated to 60 °C.

**Scheme A1:**
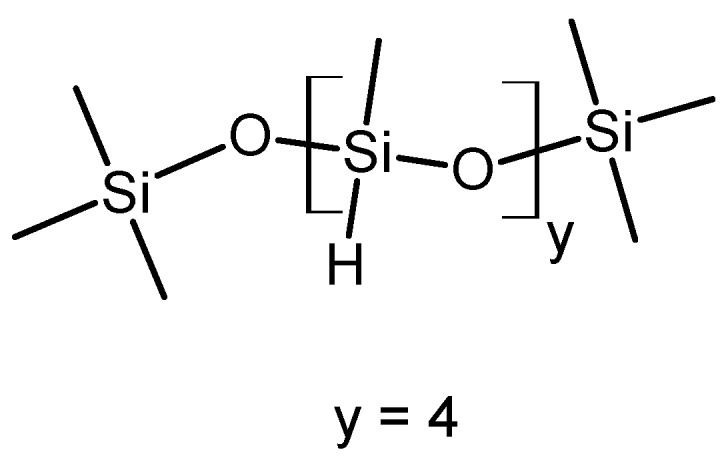
Structure of poly(methylhydrosiloxane) trimethylsilyl-terminated (PMHS).

**Scheme A2:**
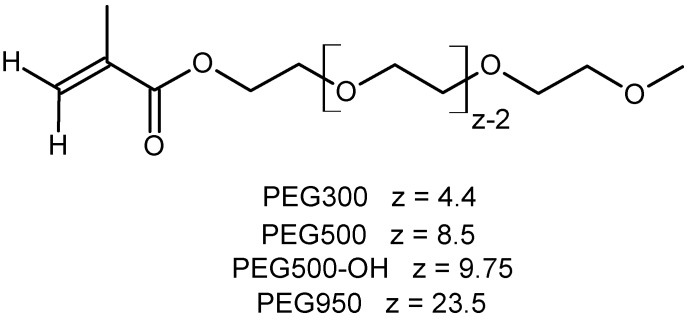
Structure of poly(ethylene glycol) methyl ether methacrylate (PEG).

After that, PMHS (Scheme A1) and Karstedt’s catalyst (PTDD) were added with a syringe through septum. The reaction was carried out for 8 h and then the PEG co-monomers (Scheme A2) were added. The process was continued for 16 h. The crude product was purified by continuous extraction using consecutively: methanol, hexane, and chloroform.

The chloroform fractions of obtained copolymers were used for spectroelectrochemical investigations. Details of the copolymers are given in Table 1.

### 2.3. Molecular Characterisation

^1^H-NMR analysis of products was performed for solutions in CDCl_3_ on a Varian Unity Inova (Palo Alto, CA, USA) spectrometer with a resonance frequency of 300 MHz using TMS as an internal standard. IR spectroscopy was carried out on a Perkin-Elmer Spectrum Two (Waltham, MA, USA) spectrometer, equipped with a universal attenuated total reflectance UATR (Single Reflection Diamond) module. The number average molecular weights (*M*_n_) and dispersities (*Đ*_M_) were determined for the THF solutions of sample concentration 1 mg/cm^3^ using a size-exclusion (SEC) 1100 Agilent (Santa Clara, CA, USA) 1260 Infinity isocratic chromatograph with a differential refractometric MDS RI Detector. The molecular weight obtained by SEC was based on calibration with linear polystyrene standards (580–300,000 g/mol).

### 2.4. Electrochemical, Spectroelectrochemical, and Conductometric Measurements

Solutions for electrochemical and spectroelectrochemical investigations were prepared by dissolving the polymer sample in chloroform (Sigma-Aldrich, CHROMASOLV, >99.9%, HPLC grade). Thin solid films on different working electrodes were prepared through spin-coating the polymer solution, typically at a concentration of 2.6 mg/cm^3^ onto an electrode.

Electrochemical and conductometric investigations were performed using a standard three-electrode cell, with an interdigitated array electrode (Pt–IAE) comprising two working electrodes (Pt path width of 5 μm and path spacing of 5 μm, produced by Dropsens (Herisau, Switzerland)), an Ag pseudo-reference electrode, and a Pt coil counter-electrode. Supplementary electrochemical (CVs) data was acquired for layers produced by drop-casting the copolymer solutions onto a Pt wire working electrode.

Measurements were carried out on a Metrohm-Autolab PGSTAT302N (Herisau, Switzerland) potentiostat equipped with a bipotentiostat module. Conductometric measurements were realised in cyclic voltammetric mode, scanning the potential, while maintaining a constant potential offset (0.005 V) between the two working electrodes.

UV-Vis-NIR spectroelectrochemical experiments were carried out in a 2 mm Hellma QS cuvette, equipped with an ITO/glass working electrode, an Ag pseudo-reference electrode, and a Pt mesh counter electrode. UV-Vis-NIR spectra were registered using an Ocean Optics (Largo, FL, USA) diode-array spectrometer set (QE65000 and NIRQuest 512). Optical contrast values were calculated using the transmittance of the polymer films in their most highly-doped and undoped states; these values were then averaged across all wavelengths in the spectral range of the spectrometer.

A solution of 0.1 M tetrabutylammonium tetrafluoroborate (Sigma-Aldrich, >99.0%, electrochemical analysis grade) in acetonitrile (Sigma-Aldrich, CHROMASOLV, >99.9%, HPLC grade) was used in all experiments as the supporting electrolyte. Prior to each measurement, each investigated sample was purged with inert gas, and the same gas was passed through the electrochemical cell during the measurement.

Applied potentials in all of the electrochemical experiments were calibrated versus the ferrocene/ferrocenium redox couple, as presented. In the case of spectroelectrochemical experiments, the applied potentials refer to the silver pseudo-reference electrode system. The silver pseudo-reference electrode was calibrated against ferrocene/ferrocenium redox couple, whose recorded potential versus this electrode was constant, amounting to +0.46 V.

### 2.5. Solar Cell Fabrication and Investigation

Photovoltaic devices with ITO/PEDOT:PSS/SilPEG:PC61BM/Al structure were prepared by spin-coating of poly(3,4-ethylenedioxythiophene):poly(4-styrenesulfonate) (PEDOT:PSS) (M121, Ossila) films on ITO Glass Substrates (Ossila (Sheffield, UK) 6 pixels) at 5000 rpm for 40 s (30 nm). PEDOT:PSS films were annealed at 140 °C for 10 min. The solutions of SilPEG and PC_61_BM (Solenne, 99.5% (Groningen, The Netherlands)) in chlorobenzene (Acros Organics, 99+ (Geel, Belgium)) solutions with 2:1 ratio and a total concentration of 25 mg/mL were filtered (Syringe Filters, 0.45 μm, PTFE membrane) at temperature of 60 °C directly before application. Active layers were spin-coated at 2000 rpm for 40 s on top of the PEDOT:PSS films, with a substrate temperature of 60 °C, and then dried at 130 °C for 10 min. Al electrodes (100 nm) were deposited on top of the active layers by thermal evaporation. The area of the device was 0.045 cm^2^. IV-curves of photovoltaic devices were measured by a PV Test Solutions Solar Simulator and a Keithley 2400 (Tektronix, Inc., Beaverton, OR, USA) in a glove box without encapsulation of devices.

## 3. Results and Discussion

### 3.1. Material Identification

Regioregular poly(3-hexylthiophene) functionalised with a terminal vinyl group was synthesised via the GRIM method, with in situ end group attachment. Polysiloxane-graft-(P3HT; PEG) samples were prepared via a variation of the hydrosilylation reaction, in the course of a facile one-pot synthesis.

**Figure 1:**
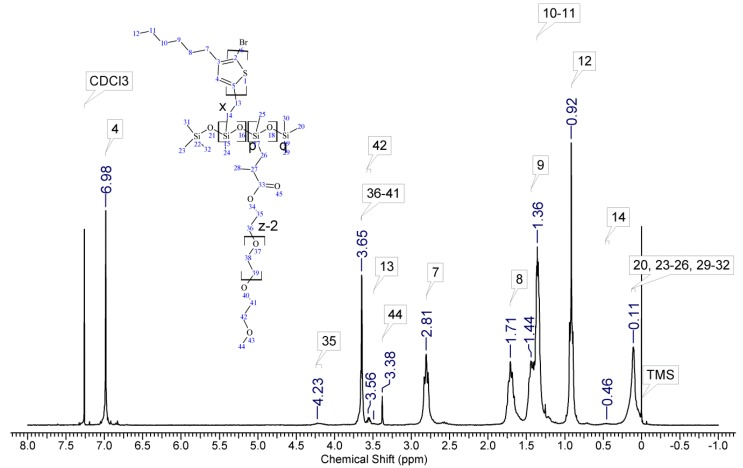
^1^H-NMR spectrum of poly(methylhydrosiloxane)-graft-[poly(3-hexylthiophene); poly(ethylene glycol) methyl ether methacrylate] (PEG *M*_n_ ≈ 500) (SilPEG 1.2).

**Figure A1:**
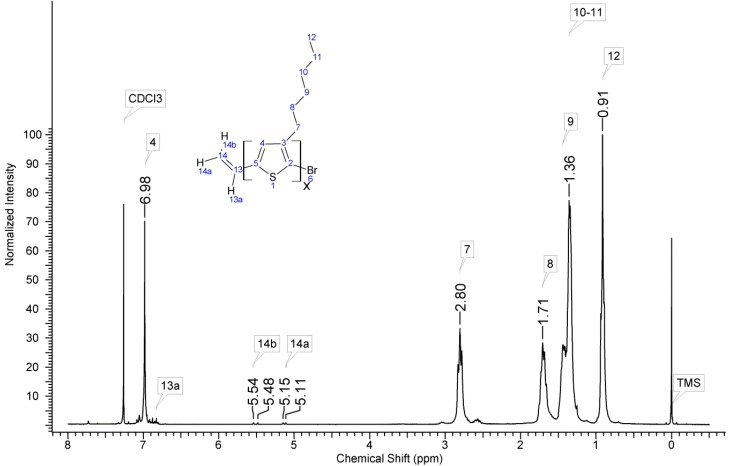
^1^H-NMR spectrum of vinyl-terminated regioregular P3HT (P3HTvin).

**Figure A2:**
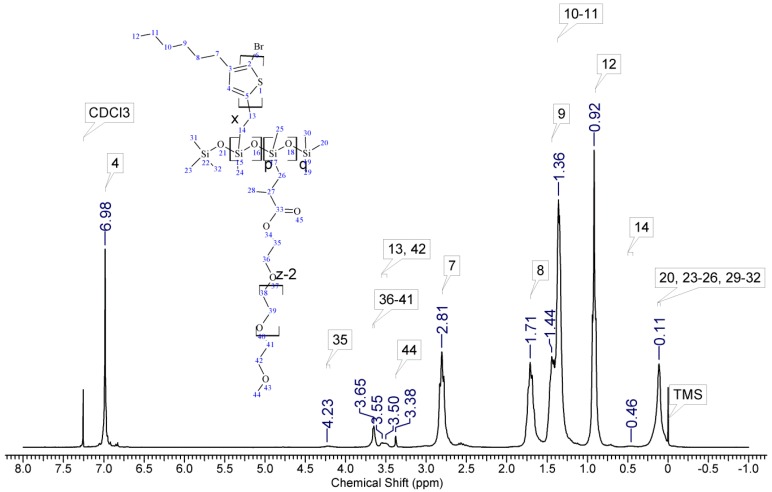
^1^H-NMR spectrum of poly(methylhydrosiloxane)-graft-[poly(3-hexylthiophene); poly(ethylene glycol) methyl ether methacrylate] (PEG *M*_n_ ≈ 300) (SilPEG1.1).

**Figure A3:**
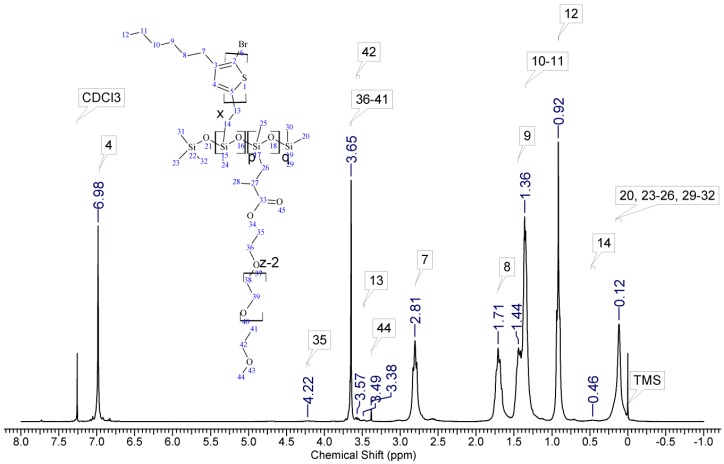
^1^H-NMR spectrum of poly(methylhydrosiloxane)-graft-[poly(3-hexylthiophene); poly(ethylene glycol) methyl ether methacrylate] (PEG *M*_n_ ≈ 950) (SilPEG1.3).

**Figure A4:**
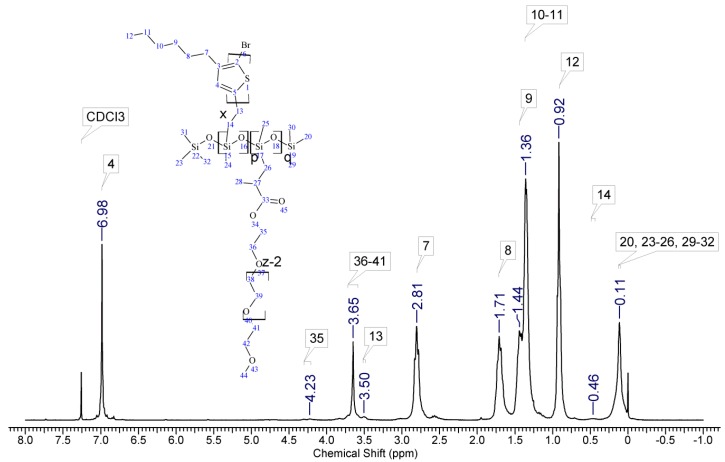
^1^H-NMR spectrum of poly(methylhydrosiloxane)-graft-[poly(3-hexylthiophene); poly(ethylene glycol) methacrylate] (PEG-OH *M*_n_ ≈ 500) (SilPEG1.4).

Structures of obtained copolymers were confirmed by comparative analysis of ^1^H-NMR spectra of reagents and final products. A demonstrative spectrum of SilPEG 1.2 copolymer is presented in the Figure 1 (the others (Figure A2, Figure A3 and Figure A4), as well as the spectra and ^1^H-NMR characteristics of P3HTvin (Figure A1), PMHS, and PEGs are included in Appendix A).

On the spectrum of P3HTvin, the signals of the geminal protons in vinyl terminal group (two doublets at δ 5.51: H14b, *J*_H14b-H13a_ = 17.8 Hz and 5.13 ppm: H14a, *J*_H14a-H13a_ = 11.4 Hz) were clearly visible; the signal of the third proton in the vinyl group, (dd δ 6.84 ppm) overlaps with the group of aromatic protons signals. On the other hand, the PMHS spectrum features the signal of hydrogen atoms attached directly to the silicon atom in the polysiloxane main chain (4.67–4.72 ppm), and the PEG spectra feature signals of protons in methacrylate group at δ 6.13 and 5.58 (**HH**C=C(CH_3_)–(C=O)O) and also at δ 1.95 ppm (=C(C**H**_3_)–(C=O)O). All these signals decayed as a result of P3HT and PEG grafting on the PMHS backbone. A series of new signals arose, namely, signals resulting from grafting P3HT on PMHS: broad signal at δ 0.46 ppm (Ar–CH_2_–C**H**_2_–Si), and at δ 3.48–3.50 ppm (Ar–C**H**_2_–CH_2_–Si). At the same time, the signal of C**H**_3_–Si protons in PMHS at 0.02–0.25 ppm broadened. All of the copolymer spectra also featured the rest of the expected signals from P3HT, PMS, and PEGs.

**Figure A5:**
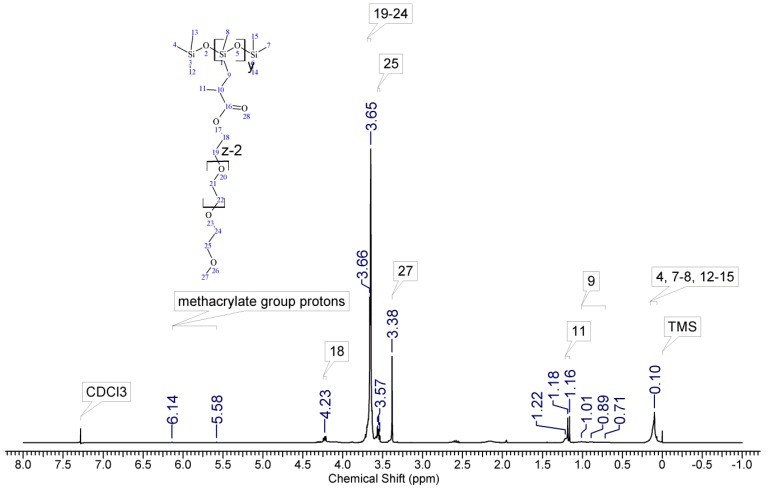
^1^H-NMR spectrum of poly(methylhydrosiloxane)-graft-[poly(ethylene glycol) methyl ether methacrylate] (PEG *M*_n_ ≈ 500) (SilPEG0).

What is more, grafting reaction was performed only with PEG. Conversion of the methacrylate group calculated based on the ^1^H-NMR spectrum of poly(methylhydrosiloxane)-graft-[poly(ethylene glycol) methyl ether methacrylate] copolymer (SilPEG 0) reaches 96% (see Figure A5 in Appendix A).

Table A1 summarises the grafting densities of the P3HT and PEG chains on the PMHS backbone calculated from ^1^H-NMR analyses, and compiled with P3HT grafting density calculated, based on feed.

**Figure 2:**
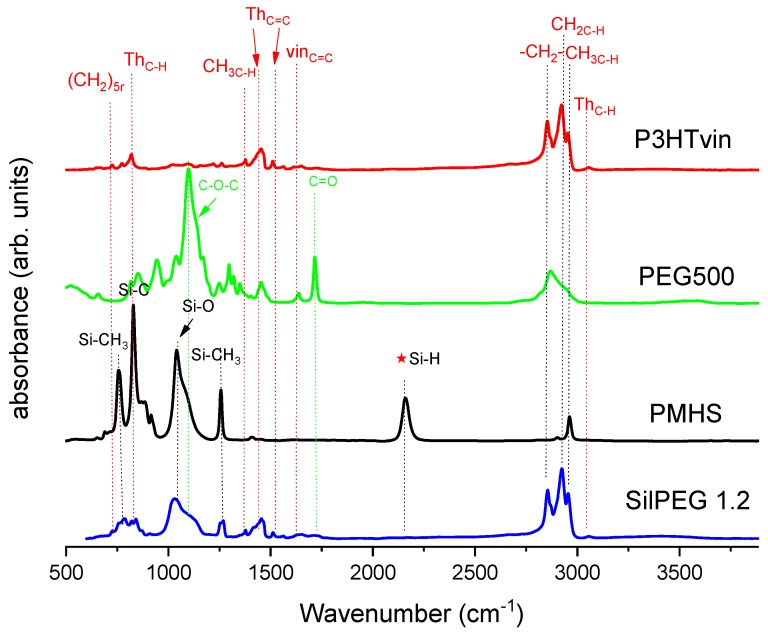
IR-ATR of poly(methylhydrosiloxane)-graft-[poly(3-hexylthiophene); poly(ethylene glycol) methyl ether methacrylate] (PEG average *M*_n_ ≈ 500) (SilPEG 1.2).

On the Figure 2, the ATR-IR spectra of the co-monomers and the graft polymer SILPEG 1.2 are presented.

**Figure A6:**
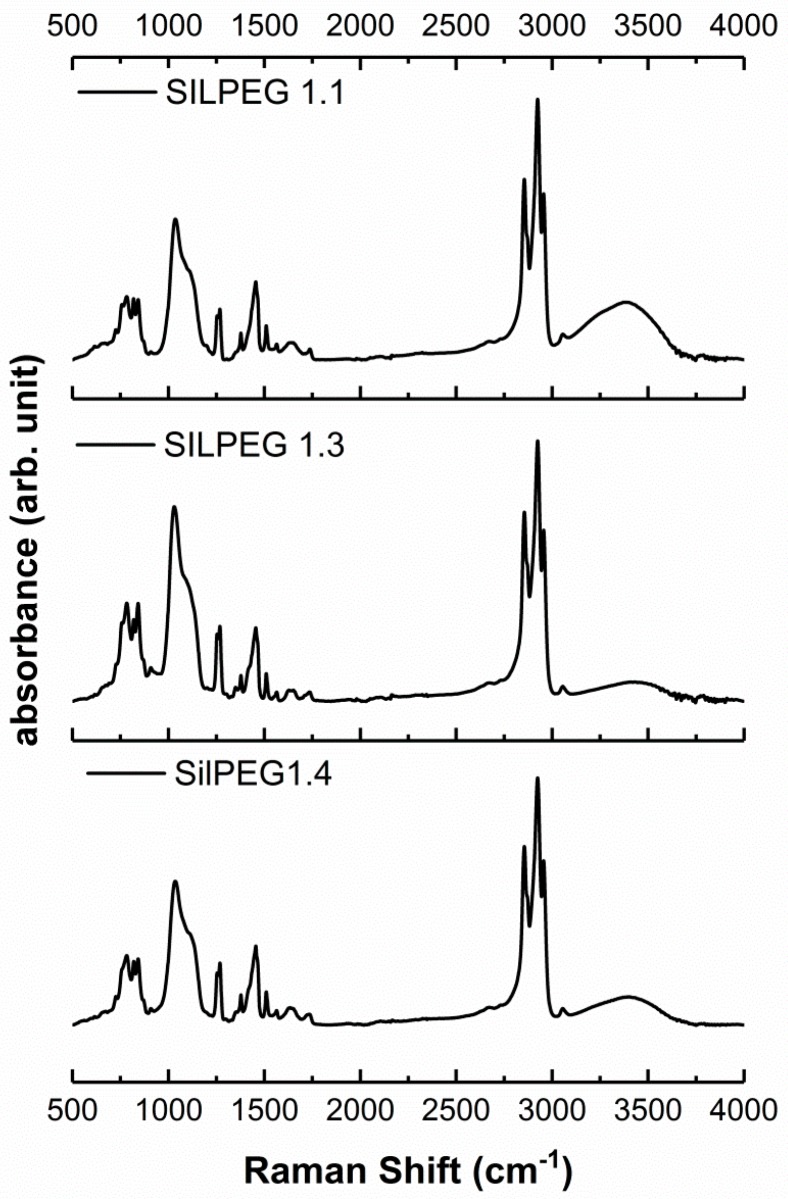
IR-ATR spectra of SilPEG1.1, SilPEG1.3, SilPEG1.4.

The IR spectra of other copolymers are similar to SilPEG1.2, and they are included in the Appendix A (Figure A6). The proposed IR spectral assignment for P3HTvin and SilPEG1.2 is shown in Table 2.

The spectrum of PEG (Figure 2) shows the characteristic vibration bands of the CH_2_ and CH_3_ framework, stretching at 2883.5 cm^−1^, bending CH_2_ and CH_3_ at 1467.8 cm^−1^, and at 1342.4 cm^−1^, respectively, as well as C–O–C asymmetric and symmetric stretching at 1103.3 cm^−1^ and at 960.5 cm^−1^, respectively. The presence of a vinyl end group was also evidenced by the C=C stretching vibration found at 1641 cm^–1^.

The Si–H peak found for PMHS at 2160 cm^−1^ and the signal of the vinyl groups at 1641 cm^−1^ from P3HTvin completely disappeared after hydrosilylation. Moreover, signals of Si–CH_2_–R wagging vibration at 1252 cm^−1^ appeared, as shown in Figure 2. The spectra of grafted copolymers not only displayed all of the characteristic peaks of siloxane groups Si–O–Si at 1090 and at 1020 cm^−1^, as well as those of P3HT, but also showed the absorption bands of poly(ethylene glycol), such as CH_2_ and CH_3_ bending at 1467.8 cm^−1^ and at 1342.4 cm^−1^, C–O–C (see Figure A6 in Appendix A). This indicates that P3HT and poly(ethylene glycol) were successfully grafted onto the polysiloxane chain.

### 3.2. Cyclic Voltammetry

**Figure 3:**
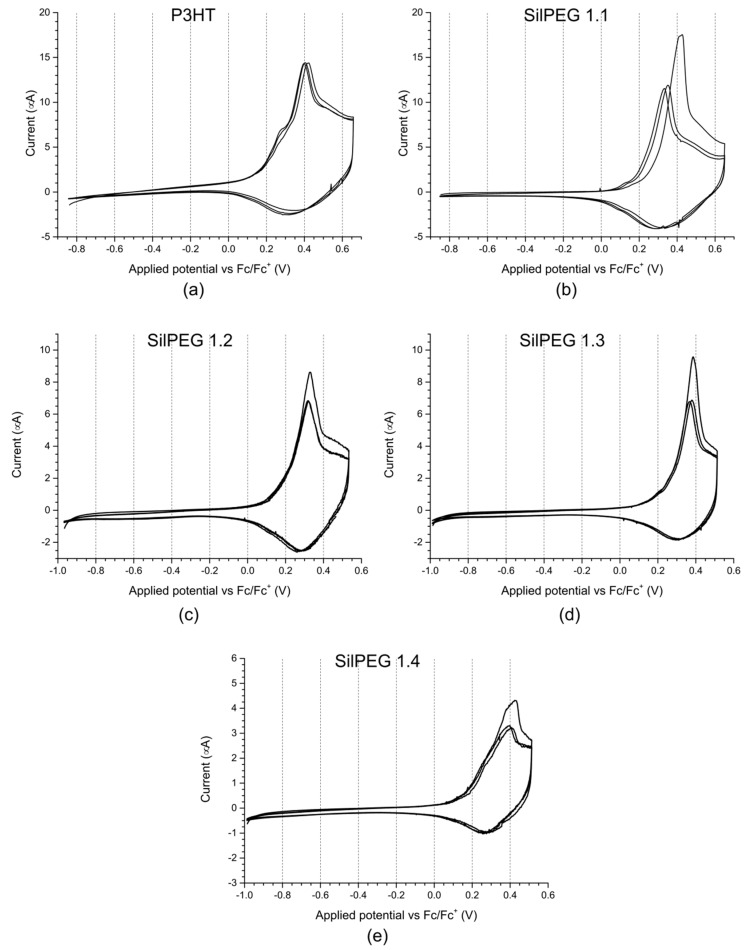
Electrochemical response of thin solid films of (**a**) regioregular P3HT; (**b**) SilPEG 1.1; (**c**) SilPEG 1.2; (**d**) SilPEG 1.3; (**e**) SilPEG 1.4 in 0.1 M tetrabutylammonium tetrafluoroborate/acetonitrile. Potential scanning rate *r* = 0.1 V/s.

The electrochemical response of regioregular poly(3-hexylthiophene) films on a platinum electrode was observed at a potential scanning rate of 0.005 V/s (Figure 3a). Interestingly, instead of the two redox pairs often seen for P3HT, two oxidation peaks are observed at +0.28 V and at +0.40 V, and a single, broad reduction band is seen at +0.33 V. The shape of this reduction signal implies that dedoping of the highly-doped polymer takes place at a limited rate, resulting in broadening of the response and shifting its apparent maximum potential towards less positive potentials, as well as obscuring the less pronounced reduction signal expected as a counterpart to the +0.28 V oxidation peak.

Upon repeated cycling, the +0.28 V oxidation peak merged into the slope of the +0.40 V signal, whose maximum potential shifted slightly towards more positive potentials. No decline in peak current was observed, evidencing that if polymer deactivation is taking place, its magnitude is negligible. Instead, such changes in the CV shape implied that film reorganisation was taking place upon doping/dedoping.

**Figure A7:**
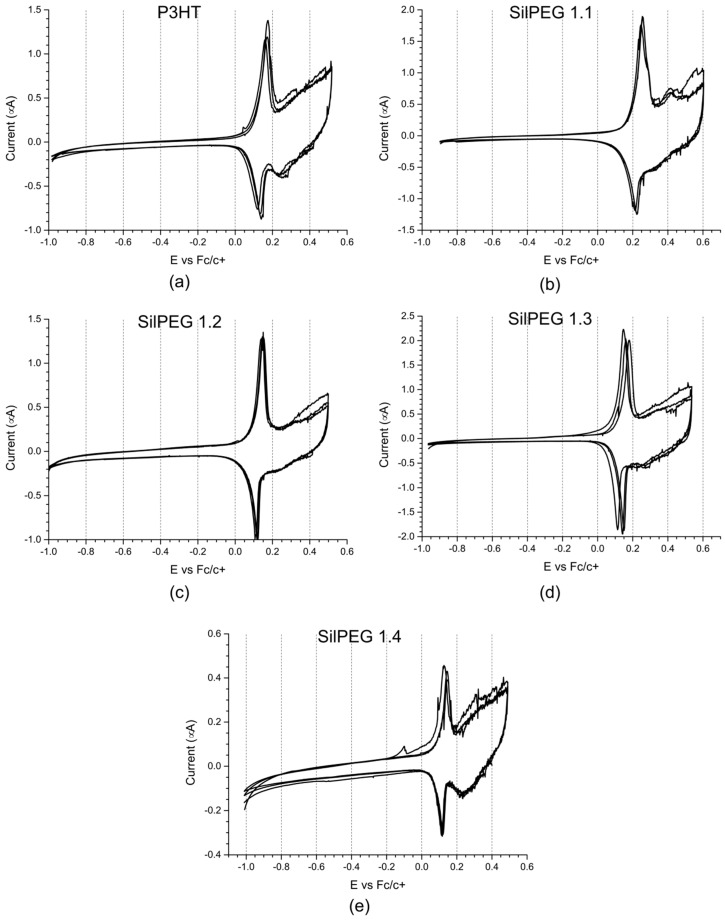
Electrochemical response of thin solid films of (**a**) regioregular P3HT; (**b**) SilPEG1.1; (**c**) SilPEG 1.2; (**d**) SilPEG 1.3; (**e**) SilPEG 1.4 deposited on a Pt wire electrode, in 0.1 M tetrabutylammonium tetrafluoroborate/acetonitrile. Potential scanning rate *r* = 0.005 V/s.

The potentials of the oxidation and reduction signals were well within the ranges reported [23,24,25,26] for P3HT and other 3-alkylthiophene polymers. To allow for comparison with most reported works, we acquired the CVs of the copolymers deposited on a Pt wire working electrode (Figure A7). Interestingly, the peak/composite signal potentials, observed in those experiments (Table 3), were very similar to what is typically found for P3HT.

In the case of SilPEG 1.1 (Figure 3b), the low potential signal was initially poorly developed and is found at +0.17 V. Upon repeated cycling it became slightly more pronounced, and it shifted towards less positive potentials. The high potential oxidation peak was found at +0.42 V. Similarly to SilPEG1.3, its peak current declined sharply between the first and second potential cycle, stabilising in subsequent cycles. Unlike SilPEG 1.3, however, its position shifted to less positive potentials, implying reorganisation of the polymer and an increase in its effective conjugation length, rather than deterioration of the polymer chains.

The shape of the CV curves recorded for SilPEG1.2 films (Figure 3c) was similar to that of regioregular P3HT films. The first oxidation peak was less pronounced and it was observed at +0.21 V, whereas the second peak was seen at +0.40 V, as was the case for P3HT. The reduction signal appeared to be slightly broader, although it was still found at +0.33 V. Upon cycling the applied potential, the oxidation peak current declined sharply in the second cycle, deteriorating only slightly in the subsequent cycles. Simultaneously, the potential of the peak shifted towards more positive potentials, with both changes being a sign of progressing deterioration of the copolymer film.

The CV curve recorded for SilPEG1.3 (Figure 3d) shows two minor oxidation signals, +0.28 V and at +0.36 V, as well as the distinct peak at +0.43 V. The increased potential of the pronounced peak (+0.40 V for P3HT) implied that the presence of the longer PEG chains slightly hinders conjugation. The occurrence of the two smaller oxidation signals was consistent with our earlier findings, and it has been attributed to the existence of ordered and amorphous phases in the polymer film. Interestingly, while this was expected to be the case in all investigated films, the signals were separate and distinct rather than overlapping. This separation implies that the two phases differed more than in the case of other investigated copolymers, implying some degree of interaction between the P3HT and long PEG *co*-grafts.

The electrochemical response of SilPEG 1.4 (Figure 3e) was particularly interesting among the investigated systems, as it consisted of a clearly composite oxidation signal, with the individual peaks at +0.38 V and at +0.43 V, and a broad reduction peak, centred at +0.27 V. In subsequent cycles, the intensity of the component signal at +0.43 V changed. This resulted in a shift in the apparent potential of the composite signal. Such behaviour implies that the polymer is undergoing reorganisation, induced both by doping and dedoping, with apparently no stable structure. Much as in the case of the two copolymers above, the response of the copolymer diminished significantly between the first and second cycle, but it remained stable in subsequent cycles, even though its shape kept shifting.

### 3.3. UV-Vis-NIR Spectroelectrochemistry

**Figure 4:**
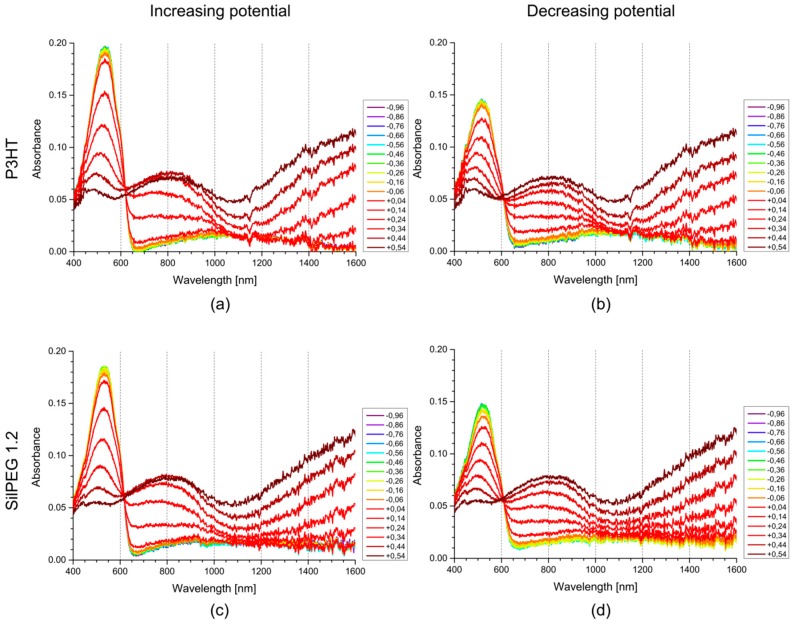
UV-Vis-NIR spectra recorded during the application of stepwise increasing (left) and decreasing (right) potential (vs. Ag/Ag+) to films of the investigated polymer and copolymer: (**a**,**b**) regioregular P3HT; (**c**,**d**) SilPEG1.2 deposited on ITO/quartz electrodes.

**Figure A8:**
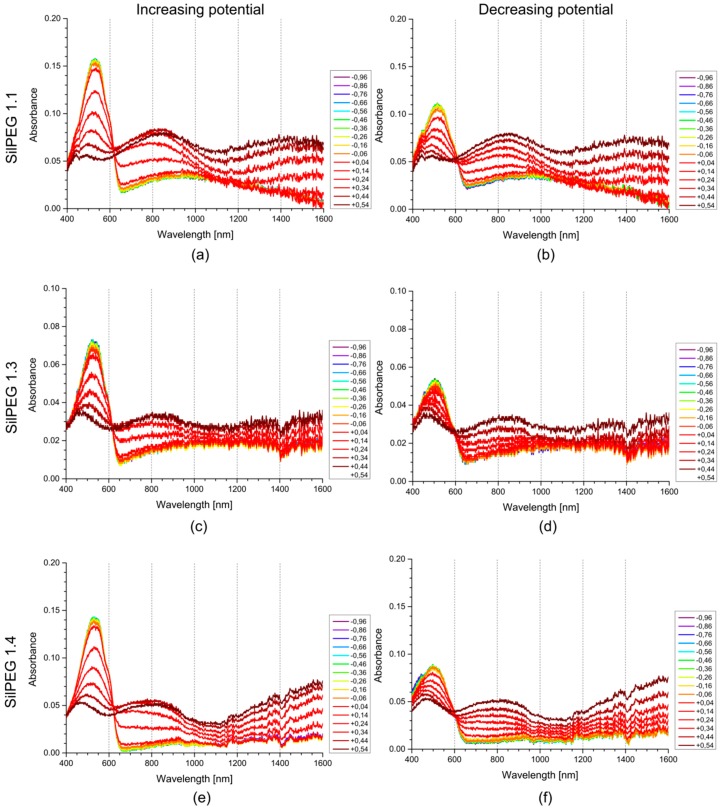
UV-Vis-NIR spectra recorded during the application of stepwise increasing (left) and decreasing (right) potential (vs. Ag/Ag+) to films of the investigated polymer and copolymer: (**a**,**b**) SilPEG 1.1; (**c**,**d**) SilPEG 1.3; (**e**,**f**) SilPEG 1.4 deposited on ITO/quartz electrodes.

The UV-Vis-NIR spectrum (Figure 4a) of the non-polarised regioregular P3HT film on ITO/quartz consisted of a broad absorption signal, with a slope at 645 nm, corresponding to an optical band gap of 1.92 eV. This value was slightly larger than what we have observed in our earlier works for drop cast films (slope at 655 nm, band gap of 1.89 eV). While the deposition method and the lack of exposure to oxygen are both expected to affect the polymer (respectively yielding a different morphology and a lack of residual doping states in the polymer film), their contributions to the slight elevation of the band gap are difficult to evaluate. Much as in our earlier works, the undoped polymer absorption signal consists of several overlapping signals, two of which are seen at 494 nm (more pronounced when positive potentials are applied to the polymer) and 599 nm, and a third, whose absorption maximum is estimated to be at 532 ÷ 537 nm. Although we first suspected these signals to arise due to vibronic structure, it was been found not to be the case [18]. The individual signals arise due to the existence of structures exhibiting long-range ordering alongside less ordered structures, showing respectively longer and shorter effective conjugation lengths in the π-bond system of P3HT. The same composite nature of the absorption signal of the undoped copolymer is seen in the spectra of all investigated graft copolymers (Figure 4c, Figure A8a,c,e). Although their positions and shares in the composite signals vary, the three individual signals were present, regardless of the length of the non-conjugated PEG *co*-grafts, or even of the use of hydroxyl-terminated PEG *co*-grafts. This implies either that the ordered P3HT domains could be formed by individual P3HT chains folding upon themselves, or that P3HT chains were grafted non-uniformly on the polysiloxane chains during synthesis, yielding P3HT-rich and PEG-rich polysiloxane chain fragments alongside the fragments where the two *co*-grafts are mixed more evenly.

Upon applying increasingly positive potentials to the P3HT film (Figure 4a), the absorption signal of the undoped polymer began to lose intensity and a slope began to evolve, starting at 1200 nm and continuing well into the NIR region. Simultaneously, a shoulder in the range of 675 ÷ 950 nm, which, on the application of +0.34 V, developed into a broad peak, was centred at 820 nm. Both the existence of this shoulder and the significant breadth of its evolved peak imply the coexistence of several doped states, differing slightly in their effective conjugation length, are formed in the polymer film. This is in line with the composite nature of the undoped polymer signal, with the oxidation of the thiophene segments, located in the ordered and amorphous domains of the polymer film, being expected to yield doped states interacting with the neighbouring chains to different extents. Interestingly, of the signals comprising the composite undoped polymer peak, only a trace signal at approx. 480 nm remained, possibly related to either the amorphous-phase or defected (in terms of regioregularity or linkage) P3HT segments.

When the potentials applied to the polymer film were being lowered (Figure 4b), the changes of the polymer spectrum reverted to a qualitatively identical spectrum as prior to the experiment. From a quantitative standpoint, it is worth noting that the absorbance of the undoped polymer peak was not fully restored (0.17 prior to the experiment, as opposed to 0.12 after the applied potential is lowered to −0.5 V), indicating partial deactivation of the polymer film over the course of the experiment.

Interestingly, in the case of SilPEG 1.2 (Figure 4c), we observed virtually the same changes as in the case of P3HT. The main observed difference is that for SilPEG 1.2, the loss of undoped polymer absorbance was initially slightly more pronounced than for P3HT at the same potentials. Upon the application of highly positive potentials (+0.54 V) the residual undoped polymer signal was observed at a minimally higher wavelength (approx. 490 nm) than for P3HT.Both these phenomena were in line with SilPEG1.2, showing a minimally lower oxidation potential, and therefore, a slightly larger effective conjugation length, than P3HT.

Upon reversing the direction of potential changes (Figure 4d), we observed the qualitative restoration of the initial absorption spectrum of the undoped copolymer. From a quantitative viewpoint, much as in the case of P3HT, we saw a significant loss of undoped polymer absorption, indicative of partial deactivation of the polymer film.

In the case of the other three copolymer films, very similar absorption changes were observed (Figure A8). As such, we opted to enclose the relevant spectra as Supplementary Information, while only summarising their key spectral parameters. As such, the individual spectral parameters of the investigated films of the different polymers are compiled in Table 4.

### 3.4. Conductance of Polymer Films

**Figure 5:**
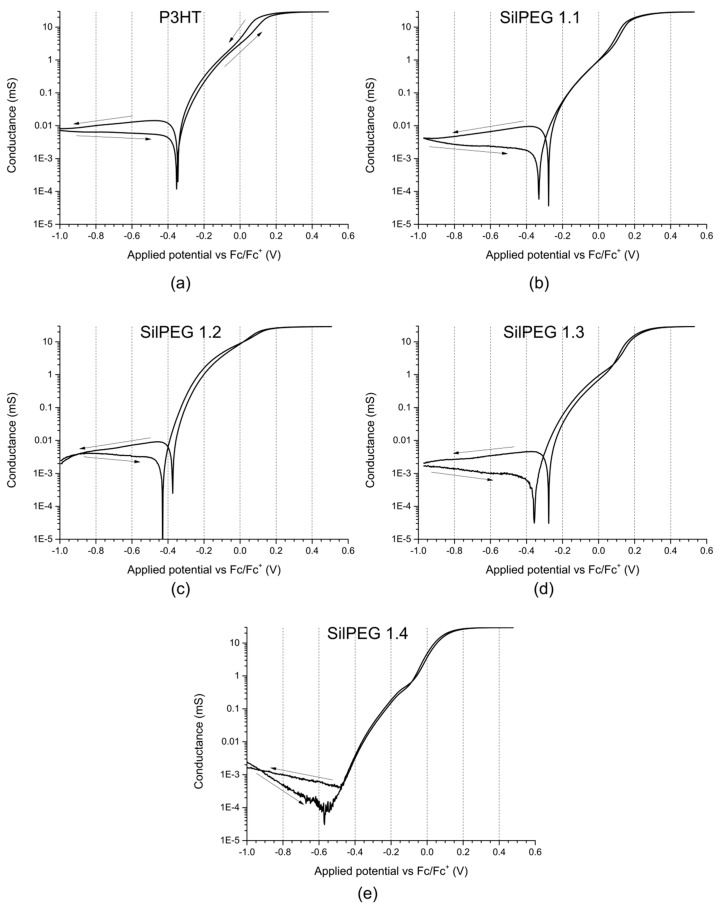
Conductance of (**a**) P3HT; (**b**) SilPEG1.1; (**c**) SilPEG1.2; (**d**) SilPEG 1.3; (**e**) SilPEG 1.4.

The conductance of regioregular P3HT (Figure 5a) was marginal at low applied potentials. This is expected, as for this study, we stored all of the prepared samples in strictly oxygen-free conditions, as opposed to light vacuum conditions in our prior work, which might have affected their initial doping states. These storage conditions were designed as a countermeasure to the possibility of P3HT doping via reactions with oxygen in the atmosphere, a known phenomenon [27].

**Figure A9:**
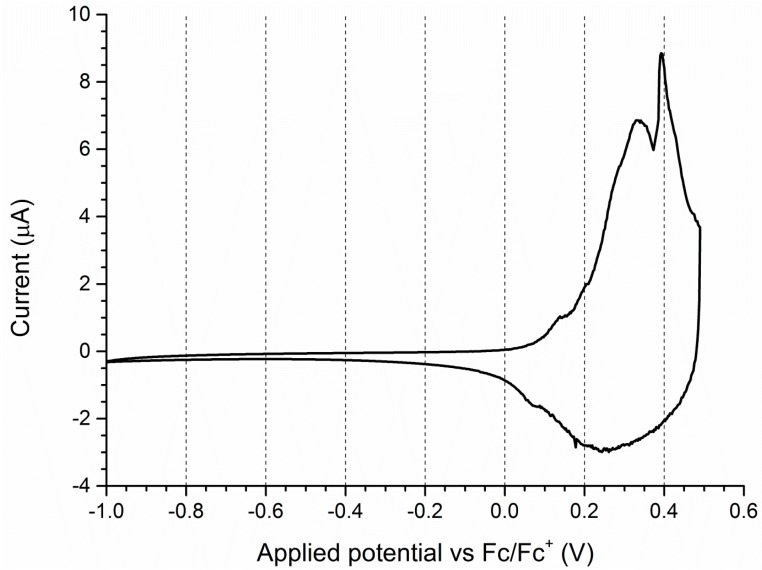
Pseudo-CV curve of P3HT recorded simultaneously with the conductance measurement.

Initially, when the potential applied to the polymer film was increased, the conductance of the polymer decreased very slowly, followed by a sharp dip in conductance at −0.35 V. This was possibly due to the removal of trace charge carriers present in the polymer film, as well as due to interactions with the supporting electrolyte, as this is the first time the polymer film was polarised electrically. As the applied potential further increased, conductance began to sharply increase, as the first charge carriers were being injected onto the P3HT chains. The conductance value kept increasing, until roughly +0.1 V, where it began to stabilise. Shortly after, at above +0.2 V, the highest value of 29.2 mS was achieved and maintained at higher potentials. Interestingly, the sharp conductance increase coincided neither with any observed CV current signals (Figure 3a) nor with the bipotentiostatic pseudo-CV current signals (Figure A9). Although discrepancies with the typical CV results could be explained by potential standardisation errors, the same could not explain discrepancies with the pseudo-CV, as it was recorded in the same experiment as the conductance values. This unexpected behaviour might possibly be explained by an extreme broadening of the current response of the first oxidation stage of the P3HT chains. Such an explanation is, however, unlikely, as the onset of the first oxidation signal of the polymer was far above 0 V. Furthermore, no visible spectral changes were observed until potentials above −0.1 V were applied to the polymer film. Consequently, it would appear that even an initially tiny population of charge carriers can yield a sharp increase in the observed conductance, while being insufficient to result in noticeable changes to the absorption spectrum.

The different rate at which conductance increases, between +0.1 V and +0.2 V, however, is believed to result from increasing interactions between charge carriers, resulting in a charge carrier “over-saturation” of the polymer and resultant loss of their mobility. This explanation is supported by the fact that the UV-Vis-NIR signals, arising from oxidised polymer segments, keep increasing up until +0.54 V is applied to the polymer film. If the population of the charge carriers keeps increasing and the observed conductance is constant, the mobility of those charge carriers must be decreasing accordingly.

Upon reversing the scan polarity (cathodic half-cycle), the conductance of the polymer was maintained until the applied potential is below +0.2 V, at which point it began to slowly decrease. A slight conductance hysteresis was observed, which was expected, as doping state hysteresis is a well-known feature of P3HT films; in fact, the observed conductance hysteresis was lower than was expected and observed in previous experiments. Once the applied potential is at approx. 0 V, conductance begins decreasing sharply, indicative of the deep removal of charge carriers from the polymer film. Repeated cycling of the applied potentials showed that while the nature of the increasing/decreasing conductance trend was relatively variable, the boundary conductance values for the “undoped” and “fully-doped” polymer were stable. This indicates good cycle-by-cycle reversibility of the polymer film, even despite its gradual deactivation, as observed in the UV-Vis-NIR spectroelectrochemical experiments.

In the case of SilPEG1.2 (Figure 5b), the conductance of the undoped copolymer film was roughly half that of the P3HT film. This was particularly interesting, seeing that not only is the share (by weight) of P3HT chains in the copolymer is much lower than for pure P3HT, but non-conjugated PEG co-grafts are also present, hindering inter-chain interactions between the grafted P3HT chains. Similar to the case of P3HT, the conductance of the copolymer film begins increasing even at strongly negative potentials, implying a similar mechanism as in the case of the P3HT film. Interestingly, the value of conductance that can be achieved for the copolymer through doping is virtually the same as that for pure P3HT. This indicates that, despite the lower concentration of P3HT in the copolymer film than for pure P3HT, a similar amount of conducting paths are formed, implying some self-organisation properties of the grafted P3HT chains.

Virtually the same conductance–potential dependence was observed for the other copolymer samples (Figure 5c–e), varying only in terms of the potential, at which conductance began increasing, and, to an extent, in the lowest observed conductance value (Table 5). The highest attainable conductance was virtually the same for each investigated copolymer sample, regardless of the P3HT content in the macromolecule. In terms of their ratio of the doped-state and undoped-state conductance (“ON/OFF” ratio), SilPEG 1.3 was found to show the highest value of 1.71 × 10^4^, evidencing that increasing the length of the PEG co-grafts is beneficial. Interestingly, the introduction of terminal hydroxyl groups appeared to be slightly detrimental to the conductance ratio for SilPEG 1.4, as SilPEG 1.2, which was equipped with PEG *co*-grafts of the same length, but lacking terminal hydroxyl groups, showed a slightly higher ON/OFF ratio.

### 3.5. Solar Cells Fabrication and Characteristics

Bulk heterojunction (BHJ) solar cells were fabricated in order to check the possibility of applying the SilPEG family of polymers in photovoltaic devices. Moreover, the influence of the polymer structure on photovoltaic performance was estimated. The conventional configuration of devices ITO/PEDOT:PSS/SilPEG:PC_61_BM/Al was chosen. The initial optimisation included the selection of the donor (SilPEG) and acceptor (PC_61_BM) weight ratios. A ratio of 2:1 was recognised as being the most efficient.

**Figure 6:**
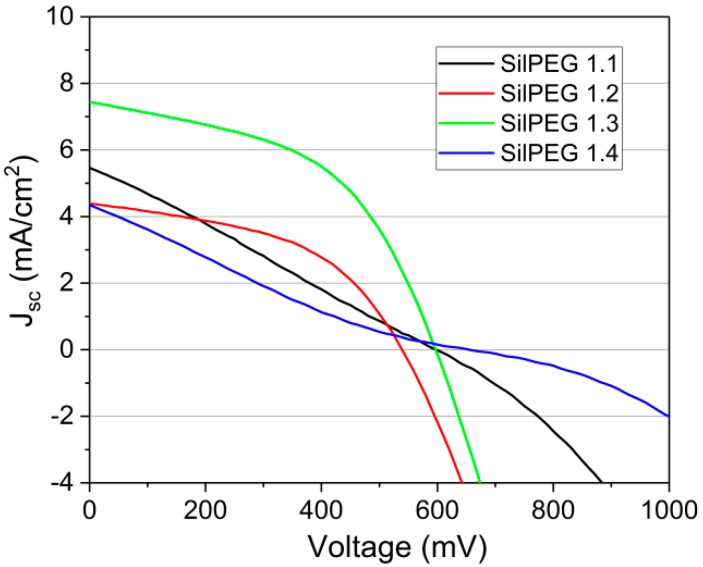
J-V characteristics of SilPEG:PC61BM blends.

Figure 6 shows the J–V curves of devices based on SilPEG blends. Results are summarised in Table 6. The analysis of device performance indicates a high impact of the PEG structure on photovoltaic performance. Surprisingly, the use of PEG chains with higher average molecular weights, *M*_n_, led to significant improvements in solar cell performance. Devices based on SilPEG 1.3 present the highest short-circuit current densities (*J*sc) of 7.4 mA/cm^2^, and the highest power conversion efficiencies (PCEs) of 2.11%.

Blends with SilPEG 1.2 were compared with SilPEG 1.4 in order to check the possible effects of the terminal group of PEG. PEG in SilPEG 1.2 chain contained a terminal methyl group, whilst PEG in the SilPEG 1.4 chain was terminated with a hydroxyl group. The comparison revealed a much higher Fill Factor (FF) of SilPEG 1.2, which resulted in a better PCE, of 1.1%. These results indicate that a slight change of polymer composition, such as the choice of terminal group, can significantly modify photovoltaic properties. This can be the result of differences in morphology and charge quenching on hydroxyl groups.

In summary, solar cells based on SilPEG and PC_61_BM blends show significant differences in photovoltaic properties, depending on the PEGs used as co-grafts. The dilution of P3HT in the obtained SilPEG polymers suggests that further optimisation of polymer structure can lead to efficient P3HT-based materials with good performance and lower contents of P3HT than conventional solar cells that are based on P3HT:PC_61_BM. Moreover, P3HT chains used for grafting are much shorter than commercially used P3HT polymers. As it is known, the polymer molecular weight of P3HT significantly affects P3HT:PCBM-based organic photovoltaic device performance [28,29]. In typical P3HT:PC_61_BM devices, high average molecular weight P3HT needs to be used to obtain efficient solar cells. Lower fractions worsen solar cell properties [30]. The obtained PCE of 2.11% was one of the highest reported values for devices based on P3HT derivatives, with the use of relatively low molecular weight chains of P3HT (*M*_n_ = 12 kDa), which used for grafting on siloxane [28,30,31,32,33]. This was another advantage of the structure of the tested polymers, in which the lower molecular weight fraction of P3HT could be used for grafting.

The obtained results are promising, and therefore further research will be made in order to improve the photovoltaic performance of the new grafted polymer structures presented. Longer PEG chains will be tested as a strong influence of its length is observed. Moreover, the detailed influence of P3HT content and the degree of grafting will be tested.

## 4. Conclusions

We have shown a synthetic pathway to a novel class of graft copolymers, in good agreement with ‘green chemistry’ concepts; the synthesis is strictly one-pot, and high yields are obtained. All of the investigated copolymers retain the major spectroelectrochemical features of their parent system, P3HT.

Interestingly, by comparing our spectroelectrochemical and conductometric results, we have noticed an early rise in the conductance of our samples, prior to the manifestation of any spectral changes. This observation shows that, unlike what was previously believed, a small population of charge carriers, insufficient for producing significant doped-state absorption or current response, may give rise to a noticeable conductance of the “undoped” polymer films.

Polymer blends based on SilPEG 1.3 show PCEs of up to 2.11%. This is a promising result, showing that the use of siloxane and PEG copolymers can lower the content of P3HT, and at the same time retain photovoltaic properties. Strong influence of the structure of PEG co-grafts indicates that proper selection of the copolymer composition is critical. Moreover, lower molecular weight fractions of P3HT (*M*_n_ = 12 kDa) can be used for grafting on siloxane.

## 5. Patents

A. Stolarczyk, R. Turczyn, A. Januszkiewicz-Kaleniak, K. Kepska, “Comb macromolecule polymethylsiloxane-graft-polymer conjugated-graft-polyether and method for producing it”, Silesian University of Technology, application submitted 2014, patent granted 2018, No. PL229564 B1.

## Figures and Tables

**Table 1:** Details of the copolymer composition.

Copolymer	Composition	PEG co-grafts	*M*_n_ and *Ð*_M_ by SEC
SilPEG 1.1	P3HTvin, poly(methylhydrosiloxane) *M*_n_ ~ 390 g/mol, poly(ethylene glycol) methyl ether methacrylate/poly(ethylene glycol) methacrylate	*M*_n_ ~ 300 g/mol Methyl-terminated	*M*_n_ = 15,000 g/mol *Ð*_M_ = 1.68
SilPEG 1.2		*M*_n_ ~ 500 g/mol Methyl-terminated	*M*_n_ = 19,000 g/mol *Ð*_M_ = 1.75
SilPEG 1.3		*M*_n_ ~ 950 g/mol Methyl-terminated	*M*_n_ = 17,000 g/mol *Ð*_M_ = 1.49
SilPEG 1.4		*M*_n_ ~ 500 g/mol Hydroxyl-terminated	*M*_n_ = 16,000 g/mol *Ð*_M_ = 1.65

**Table 2:** The IR band assignments.

Polymer	Assignment
P3HTvin	3057 (ν C_Ar_–H w), 2965 (ν_asym_ C–H s), 2928 (ν_asym_ C–H s), 2855 (ν_sym_ C–H s), 1620 (ν C=C w), 1563 (ν C_Ar_=C_Ar_ w), 1511 (ν_sym_C_Ar_=C_Ar_m), 1456 (δ_asym_ C–H[–CH_3_]m), 1374 (δ_sym_ C–H[–CH_3_, –CH_2_–]m), 1217 (νC_Ar_=C_Ar_ w), 1155 (δ C–H[–CH=CH_2_] w), 819 (δC_Ar_–H m), 724 (δ=C–H; δ –(CH_2_)_n>3_ w), 668 (δ=C–H w)
SilPEG 1.2	3057 (ν C_Ar_−H w), 2965 (ν_asym_ C–H s), 2928 (ν_asym_ C–H s), 2855 (ν_sym_C–H s), 15,656 (ν C_Ar_=C_Ar_ w), 1513 (ν_sym_C_Ar_=C_Ar_ w), 1451 (δ_asym_ C–H m), 1253 (δ_sym_ Si(CH_3_)_n=1,3,4_ m), 1028 (ν C=O s), 818 (δ C_Ar_–H m), 724 (δ = C–H; δ –(CH_2_)_n>3_ m)

* Band intensities: s—strong, m—medium, w—weak.

**Table 3:** Oxidation and reduction potentials of the investigated films deposited via drop-casting on the Pt wire electrodes.

Sample	Oxidation Potentials [V]	Reduction Potentials [V]
P3HT	+0.18	+0.23; +0.14
SilPEG 1.1	+0.25; +0.41	+0.21
SilPEG 1.2	+0.15	+0.12
SilPEG 1.3	+0.15	+0.11
SilPEG 1.4	+0.38; +0.43	+0.27

**Table 4:** Key spectral parameters for the investigated P3HT and copolymer films.

Sample	Undoped Polymer Absorbance		Band Gap ^c^ [eV]	Isosbestic Point [nm]		Oxidised Polymer Absorption Band Maxima [nm]	
	Initial ^a^	Final ^b^		Anodic ^d^	Cathodic ^e^	1st Band	2nd Band
P3HT	0.17	0.12	1.93	617	608	817	1587
SilPEG 1.1	0.20	0.15	1.92	619	606	819	1537
SilPEG 1.2	0.17	0.13	1.93	615	608	808	1586
SilPEG 1.3	0.05	0.03	1.94	615	594	828	1513
SilPEG 1.4	0.11	0.05	1.93	624	597	810	1533

^a^ Absorbance prior to the application of any electrochemical stimuli to the polymer film; ^b^ Absorbance of the polymer film after the experiment; ^c^ Optical band gap value; ^d^ Isosbestic point position when applied potential was being increased in 0.1 V steps from −1.0 to +0.5 V; ^e^ Isosbestic point position when applied potential was being decreased in 0.1 V steps from +0.5 to −1.0 V.

**Table 5:** “ON/OFF” conductance ratios for the investigated polymer films.

Polymer/Copolymer	Conductance [mS]		Doped/Undoped (“ON/OFF”) Ratio
	Undoped State	Doped State	
P3HT	7.28 × 10^−3^	2.92 × 10^1^	4.01 × 10^3^
SilPEG 1.1	4.19 × 10^−3^	2.89 × 10^1^	6.90 × 10^3^
SilPEG 1.2	2.42 × 10^−3^	2.91 × 10^1^	1.20 × 10^4^
SilPEG 1.3	1.70 × 10^−3^	2.90 × 10^1^	1.71 × 10^4^
SilPEG 1.4	2.86 × 10^−3^	2.95 × 10^1^	1.03 × 10^4^

**Table 6:** PL1. Photovoltaic parameters of the BHJ solar cells.

Copolymer	*J*sc ^a^ [mA/cm^2^]	*V*oc ^b^ [V]	FF ^c^	PCE ^d^ [%]
SilPEG 1.1	5.5	0.60	0.26	0.87 ± 0.15
SilPEG 1.2	4.4	0.54	0.48	1.10 ± 0.07
SilPEG 1.3	7.4	0.60	0.50	2.11 ± 0.02
SilPEG 1.4	4.4	0.66	0.21	0.60 ± 0.19

^a^ short-circuit current density; ^b^ open circuit voltage; ^c^ fill factor; ^d^ power conversion efficiencies.

## Appendix A

^1^H-NMR spectra:

PMHS ^1^H NMR (300 MHz, CDCl_3_) δ4.79–4.62 (m, 4H) Si–**H**, 1.58 (s, 1.2 H) water, 0.24–0.04 (m, 30 H) Si–C**H**_3_.

PEG300 ^1^H NMR (300 MHz, CDCl_3_) δ 6.13 (dq, *J* = 1.6, 0.9 Hz, 1H) **H**HC = C(CH_3_)–(C = O)O, 5.58 (p, *J* = 1.6 Hz, 1H) H**H**C = C(CH_3_)–(C = O)O, 4.30 (m, 2H) (C = O)O–C**H**_2_–CH_2_–, 3.75 (m, 2H) (C = O)O –CH_2_–C**H**_2_–[O–CH_2_–CH_2_–]n, 3.65 (m, 11H)–[O–C**H**_2_–C**H**_2_–]n, 3.55 (m, 2H) –C**H**_2_–O–CH_3_, 3.38 (s, 3H) O–C**H**_3_, 1.95 (m, 3H) = C(C**H**_3_)–(C = O)O.

PEG500 ^1^H NMR (300 MHz, CDCl_3_) δ 6.13 (dq, *J* = 1.7, 1.0 Hz, 1H)**H**HC = C(CH_3_)–(C = O)O, 5.58 (p, *J* = 1.6 Hz, 1H)H**H**C = C(CH_3_)–(C = O)O, 4.30 (m, 2H) (C = O)O–C**H**_2_–CH_2_–, 3.75 (m, 2H) (C = O)O–CH_2_–C**H**_2_–[O–CH_2_–CH_2_–]n, 3.65 (m, 28H)–[O–C**H**_2_–C**H**_2_–]n, 3.55 (m, 2H) –C**H**_2_–O–CH_3_, 3.38 (s, 3H) O–C**H**_3_, 1.95 (dd, *J* = 1.6, 1.0 Hz, 3H) = C(C**H**_3_)–(C = O)O.

PEG500-OH^1^H NMR (300 MHz, CDCl_3_) δ 6.13 (m, 1H) **H**HC = C(CH_3_)–(C = O)O, 5.58 (m, 1H) H**H**C = C(CH_3_)–(C = O)O, 4.29 (m, 2H) (C = O)O–C**H**_2_–CH_2_–,3.65 (m, 37H) –[O–C**H**_2_–C**H**_2_–]n, 2.92, 2.81 (s, 1H) –O**H**, 1.95 (m, 3H) = C(C**H**_3_)–(C = O)O.

PEG950 ^1^H NMR (300 MHz, CDCl_3_) δ 6.13 (dq, *J* = 1.5, 0.9 Hz, 1H)**H**HC = C(CH_3_)–(C = O)O, 5.58 (p, *J* = 1.6 Hz, 1H)H**H**C = C(CH_3_)–(C = O)O, 4.30 (m, 2H) (C = O)O–C**H**_2_–CH_2_–, 3.65 (m, 92H)–[O–C**H**_2_–C**H**_2_–]n, 3.38 (s, 3H) O–C**H**_3_, 1.95 (m, 3H) = C(C**H**_3_)–(C = O)O.

**Table A1:** Grafting density of P3HT and PEG in SilPEG copolymers; calculations based on ^1^H-NMR analyses.

Copolymer	n P3HT per PMS Molecule	n PEG per PMS Molecule	Grafting Density [%]		P3HT Grafting Density by Feed [%]
			P3HT	PEG	
SilPEG 1.1	0.53	0.32	13.3	8.0	3.2
SilPEG 1.2	0.34	0.54	8.5	13.5	3.2
SilPEG 1.3	0.26	0.18	6.5	4.5	3.2
SilPEG 1.4	0.49	0.25	12.3	6.3	3.2
